# Editorial: Exploring genomic instability of cancers: applications in diagnosis and treatment

**DOI:** 10.3389/fcell.2025.1528281

**Published:** 2025-02-07

**Authors:** Dong Zhang, Shiaw-Yih Lin, Marietta Lee

**Affiliations:** ^1^ Department of Biomedical Sciences, College of Osteopathic Medicine, New York Institute of Technology, Old Westbury, NY, United States; ^2^ Center for Cancer Research, New York Institute of Technology, Old Westbury, NY, United States; ^3^ Department of Systems Biology, The University of Texas MD Anderson Cancer Center, Houston, TX, United States; ^4^ Department Biochemistry and Molecular Biology, New York Medical College, Valhalla, NY, United States

**Keywords:** genomic instability, cancer, hypoxia, lung adenocarcinoma, PARP inhbitors, tumor immunity

“Yin and Yang” is an ancient Chinese philosophical concept that describes two opposing and coexisting forces. Here, we use the “Yin and Yang” concept to illustrate the role of genomic instability in various aspects of cancers. It has been well-recognized that genomic instability is one of the key driving forces for cancer development. Together with “Tumor-promoting Inflammation,” genome instability and mutation were coined as the “Enabling Characteristics” of tumorigenesis by Hanahan and Weinberg (2011). For example, mutations in many DNA damage response (DDR) and DNA repair genes, such as *BRCA1* and *BRCA2*, facilitate or enable the development of various cancers (Couch et al., 2014; King, 2014). On the other hand, the survival of many cancers with unstable genomes tends to rely heavily on certain DDR and DNA repair pathways, which become their Achille’s Heels and thus can be targeted for cancer therapy. For example, we and others have shown that cancers adopted the Alternative Lengthening of Telomeres (ALT) pathway depend on the critical function of the FANCM complex to actively disrupt the TERRA R-loops and suppress replication stress at their telomeres (Pan et al., 2017; Lu et al., 2019; Silva et al., 2019; Pan et al., 2019). Inhibition of FANCM in the ALT+ cells causes a drastic decrease in their viability. This Research Topic further highlights the “Yin and Yang” of genomic instability with respect to cancer development and cancer therapy.

In a research paper by Abou Khouzam et al., the authors investigated how the sensitivity to hypoxia affects genome stability. Previously, the authors established an 8-gene hypoxia signature using various cancer cell lines (Abou Khouzam et al., 2020). Based on the 8-gene hypoxia signature, the authors separated cancers into two groups: 1) hypoxia-sensitive or hypoxia-high (HH) cancers; and 2) hypoxia resistance or hypoxia-low (HL) cancers. They then investigated how long-term exposure to hypoxia affects the properties of HH cancers and HL cancers. They found that chronic hypoxia induces genomic instability in HH cancers, leading to metabolic reprogramming and increased proliferation and survival.

In a separate research paper by Zhang et al., the authors identified a prognostic signature from The Cancer Genome Atlas (TCGA) database for lung adenocarcinoma, called GULPsig, which consists of 42 genome instability-related genes. They then compared GULPsig with the established prognostic signature for lung adenocarcinoma and found that the GULPsig outperformed the established prognostic signature. GULPaig can potentially be used as a novel prognostic signature to predict the treatment outcome of lung adenocarcinoma in the future.

In the last 20 years, DDR and DNA repair pathways have been explored for targeted therapies for treating various cancers (Pilie et al., 2019; Groelly et al., 2023). With the approval of the first PARP inhibitor, Olaparib (LYNPARZA, KuDOS/AstraZeneca), by the FDA in December 2014 to treat ovarian cancers with *BRCA1/2* mutation, the concept of targeting DDR and DNA repair to treat cancers has finally been validated. Ragupathi et al. reviewed and summarized the clinical outcomes of four different PARP inhibitors. In addition, they carefully reviewed the critical roles of BRCA1, BRCA2, and PARP in DNA replication and DNA repair at the difficult-to-replicate (DTR) regions of human genomes, regions such as centromeres, common fragile sites, rDNA loci, and telomeres. Based on their comprehensive literature reviews, they put forth the following hypothesis: because BRCA1, BRCA2, and PARP all play a critical role in DNA replication and DNA repair at the DTRs, mutations in *BRCA1* and *BRCA2* predispose the DTRs to heightened replication stress. Inhibition of PARP in cancer cells with the *BRCAness* further exacerbate the replication stress at the DTRs, which then leads to cell death.

Finally, Requesens et al. illustrated the “Yin and Yang” concept with respect to the crucial role of genomic instability in tumor immunity. DNA damage is often elevated in cells with unstable genomes, leading to increased broken DNA, physically separated from the main chromosomes. Some of the broken DNA can be protected in micronuclei, while others may be accidentally leaked into the cytosol where it will be detected by cytosolic DNA sensors, causing the activation of the cGAS-STING pathway. Requesens et al. first reviewed the pro- and anti-tumor response of the cGAS-STING pathway. Another consequence of cells with an unstable genome is that they produce many aberrant proteins, which may induce the immune response. Requesens et al. then reviewed various scenarios of immunogenicity in tumors. Because immune evasion is one of the key hallmarks of cancer (Hanahan and Weinberg, 2011; Hanahan and Weinberg, 2000), Requesens et al. finally reviewed how genomic instability may help tumors evade immune response. In the last 10 years, immunotherapy has revolutionized cancer treatment (Waldman et al., 2020). A better understanding of the role of DDR, DNA repair, and genomic instability in immuno-oncology may help to improve the current immunotherapy and/or develop better biomarkers for the current immunotherapy (Chabanon et al., 2021).
